# Sublethal and Transgenerational Toxicities of Chlorfenapyr on Biological Traits and Enzyme Activities of *Paracoccus marginatus* (Hemiptera: Pseudococcidae)

**DOI:** 10.3390/insects13100874

**Published:** 2022-09-26

**Authors:** Jian-Yu Li, Yan-Ting Chen, Qiu-Yue Wang, Li-Zhen Zheng, Jian-Wei Fu, Meng-Zhu Shi

**Affiliations:** 1 Fujian Key Laboratory for Monitoring and Integrated Management of Crop Pests, Institute of Plant Protection, Fujian Engineering Research Center for Green Pest Management, Fujian Academy of Agricultural Sciences, Fuzhou 350013, China; 2 Fujian Key Laboratory of Agro-Products Quality and Safety, Institute of Quality Standards and Testing Technology for Agro-Products, Fujian Academy of Agricultural Sciences, Fuzhou 350001, China

**Keywords:** *Paracoccus marginatus*, chlorfenapyr, sublethal dose exposure, transgenerational effects, enzyme activities

## Abstract

**Simple Summary:**

Papaya mealybug, *Paracoccus marginatus*, is an important invasive pest worldwide, which attacks more than 200 host plants. Chlorfenapyr has been demonstrated to have a significant control effect on *P. marginatus*. To evaluate the long-term sublethal effects of chlorfenapyr on *P. marginatus*, the sublethal and transgenerational effects of chlorfenapyr on the biological traits and changes of enzyme activities of *P. marginatus* were investigated. The results showed that chlorfenapyr had significant effects on the development of subsequent generations of *P. marginatus*, and chlorfenapyr also activated the activities of SOD of *P. marginatus*. The results demonstrated that chlorfenapyr-mediated sublethal effects occur in at least two successive generations of *P. marginatus*. Therefore, it is necessary to reapply the chlorfenapyr prior to emergence of the F_3_ generation to suppress the population and prevent outbreaks of *P. marginatus*.

**Abstract:**

Papaya mealybug, *Paracoccus marginatus* Williams and Granara de Willink (Hemiptera: Pseudococcidae), is an economically important, invasive insect that is now distributed worldwide. Chlorfenapyr has been demonstrated to have a significant control effect on *P. marginatus*. In order to evaluate the sublethal and transgenerational effects of chlorfenapyr on *P. marginatus*, the life table data of three consecutive generations were collected and analyzed by the age stage, two-sex life table method, and the enzyme activities were assayed using a spectrophotometer. The results showed that exposure to the insecticide had significant effects on the biological traits of subsequent generations of *P. marginatus*, and a higher intrinsic rate of increase (*r*), finite rate of increase (*λ*), net reproductive rate (*R*_0_), and a shorter mean generation time (*T*) were observed in the chlorfenapyr-treated F_1_ mealybugs. Enzyme activity assays showed that chlorfenapyr significantly inhibited the activities of catalase (CAT) and peroxidase (POD) while activating the activities of superoxide dismutase (SOD), which suggested that SOD, CAT, and POD may play an important role in the self-defense of *P*. *marginatus* against chlorfenapyr. These results conclusively demonstrated that exposure of *P. marginatus* to sublethal concentrations of chlorfenapyr induced hormetic effects on the F_1_ generation while having negative effects on the F_0_ and F_3_ generations.

## 1. Introduction

The papaya mealybug, *Paracoccus marginatus* Williams and Granara de Willink (Hemiptera: Pseudococcidae), is an economically important invasive pest in subtropical and tropical regions throughout the world [1,2]. *P. marginatus* is known to damage over 200 plant species from 60 families of plants [1,3], causing serious economic losses and potential threats to numerous economically important crops as well as a great number of ornamental plants [4]. Chemical control has been the primary strategy used in many integrated pest management (IPM) systems because it is often the fastest and most efficient means of pest control while providing reliable and effective control of targeted pests. Currently, chemical control application of profenophos, chlorpyriphos, buprofezin, dimethoate, imidaclopride, thiametoxam, and acetampride are the insecticides most commonly employed against the papaya mealybug in field situations in India and Sri Lanka [5,6,7]. Our previous toxicological tests showed that chlorfenapyr had the highest toxicity among the 15 insecticides we tested [8]. Chlorfenapyr, which is a member of the pyrroles class of insecticides, has a broad insecticidal spectrum that functions by disrupting the oxidative phosphorylation of the H proton gradient, resulting in the interruption of ATP and ultimately death of the organism [9,10]. At present, chlorfenapyr is used mainly in controlling *Spodoptera litura* (Fabr.) (Lepidoptera: Noctuidae) [11], *Plutella xylostella* (L.) (Lepidoptera: Plutellidae) [12], and *Tetranychus cinnabarinus* (Boisduval) (Acari: Tetranychidae) [13]. The recommended field dose range of chlorfenapyr is 90–120 g a.i. ha^−1^ based on the British Crop Production Council Pesticide Manual (version 6.0) [14].

After application of insecticides in the field, pests may be exposed to different sublethal doses of insecticides, and such sublethal doses result in different biological outcomes. For example, sublethal effects of pesticides may affect host biology in several ways, such as prolonging the duration of their developmental, reducing their survival rate and fecundity, and, consequently, disrupting the population dynamics of the targeted insects [15,16]. Previous studies have mainly reported the effects of sublethal doses on a single generation of the pest. Some insects, however, may continue to be exposed to sublethal doses for extended periods of time, which may cause transgenerational effects on their offspring [17]. For example, low lethal concentrations of acetamiprid and buprofezin were found to affect the duration of the preadult period, survival rate, reproduction, and population growth rate of *Brevicoryne brassicae* (L.) (Hemiptera: Aphididae) in the F_1_ generation [18]. The average fecundity, intrinsic rate of increase (*r*), and finite rate of increase (*λ*) of *Sogatella furcifera* (Horváth) (Hemiptera: Delphacidae) in the F_4_ generation were higher than those in the F_0_ and F_1_ generations after exposure to a sublethal dose of triflumezopyrim [19]. Instances such as these demonstrate the importance of evaluating the transgenerational effects of sublethal doses of pesticides on insects.

Insects often reduce the toxicity of pesticides via activating or inhibiting detoxification by their internal enzyme system [20,21,22]. Acetylcholinesterase (AchE) and carboxylesterase (CarE) are important detoxifying enzymes, which can metabolize the toxins in insects to maintain the physiological activities [23]. Antioxidative enzymes mainly include superoxide dismutase (SOD), catalase (CAT), and peroxidase (POD), and the combined action of these three enzymes maintains free radicals at low levels in insects to protect the cells from damage [24]. Enzyme activities can be used as a biomarker of organisms exposed to sublethal dose of insecticides [25].

In this study, we evaluated the long-term effects of sublethal exposure and transgenerational effects of chlorfenapyr on *P*. *marginatus* using the age-stage two-sex life table and assessed the detoxifying and antioxidative enzymes activities of *P. marginatus*. The results will contribute important information for the chemical control of *P. marginatus*, such as determining the most effective pesticide dosage and establishing the application interval.

## 2. Materials and Methods

### 2.1. Insects

*Paracoccus marginatus* were collected from pawpaw trees at the Fujian Agriculture and Forestry University, Fuzhou, Fujian Province, China. These insects were continuously reared on potato sprouts in laboratory without exposure to any insecticides for above 20 consecutive generations. The mealybugs were reared in an artificial climate chamber set at 28 ± 1 °C, 70 ± 5% relative humidity, a photoperiod of 14 L:10 D, and a light intensity of 12,000 l× at the Institute of Plant Protection, Fujian Academy of Agricultural Sciences.

### 2.2. Toxicity of Chlorfenapyr on P. marginatus

A 10% chlorfenapyr suspension concentrate was purchased from BASF (China) Co., Ltd. (Shanghai, China). A series of chlorfenapyr solutions with different concentrations were prepared by diluting with distilled water (2.5, 5, 10, 20, 40, 80 mg·L^−1^). Distilled water was used as the control group. Each of the treatments was replicated three times. At least thirty nymphs per replicate were used for toxicity test. Newly emerged 2nd-instar *P. marginatus* nymphs were carefully placed on a leaf disc (3 cm diameter) cut from sweet potato leaves. After two hours, the unestablished insects were removed, and the living individuals were left and used for test. The leaf discs containing the control and treatment groups of mealybugs were dipped into either water or one of the above chlorfenapyr solutions for 15 s [26,27] and then removed and allowed to dry at room temperature. After thoroughly dry, the leaf discs with the mealybugs were placed in a Petri dish (3.5 cm diameter) containing 2% (*w*/*v*) agar to retain leaf moisture. The leaf discs and mealybugs were then returned to the artificial climate chamber described previously. After 48 h, the mortality of *P*. *marginatus* was recorded.

### 2.3. Transgenerational and Sublethal Effects of Chlorfenapyr on P. marginatus

We contrasted the life history parameters of the F_0_, F_1_, and F_3_ generations from the control and treatment groups to determine the transgenerational effects of chlorfenapyr. One hundred newly oviposited eggs laid within a 24 h period of F_0_ generation were randomly selected for life table study. After eggs hatched into nymphs and grew to 2nd-instar nymphs, the nymphs were treated with LC_30_ of chlorfenapyr as described in Section 2.2. Forty-eight hours after treatment, the survivors (F_0_-chlorfenapyr) were numbered and individually transferred to new untreated sweet potato leaf discs for life table study. Until growth to adults, males and females were paired in centrifuge tubes (1.5 mL) and reared with fresh sweet potato leaves. The development duration, survival, and fecundity of the individuals were observed and recorded daily until the death of all individuals. For the F_1_ generation, one hundred eggs from F_0_ adults were randomly collected. The newly hatched nymphs were individually transferred to plastic cases (3.5 cm diameter) containing sweet potato leaves, and the growth and survival of the nymphs were observed daily. When adults emerged, they were paired in centrifuge tubes. The feeding method and the data to be recorded were the same as those for the F_0_ adults. The life table research method of the F_3_ generation was the same as that for the F_1_ generation. All experiments were conducted in the artificial climate chamber described above.

### 2.4. Enzyme Activity Assay

In this experiment, activities of five enzymes, AChE, CarE, CAT, SOD, and POD, were assayed. There were two treatments of each enzyme: F_0_-chlorfenapyr and F_0_-CK. Additionally, there were three replicates per treatment for each enzyme. The 2nd-instar nymphs of *P. marginatus* were treated with chlorfenapyr at concentrations of LC_30_ for 48 h, and the survivors (F_0_-Chlorfenapyr) were picked up. The control groups (F_0_-CK) were treated with distilled water. Then, 8 mg of survivors of each replicate were collected in a 1.5 mL centrifuge tube and treated with liquid nitrogen and stored in −80 °C. According to the kit instructions, the absorbance of AChE, CarE, CAT, SOD, and POD were read at 412 nm, 450 nm, 405 nm, 550 nm, and 420 nm using a spectrophotometer (TU-1900, Beijing Purkinje General Instrument Co., Ltd., Beijing, China), respectively.

### 2.5. Data Analysis

The LC_30_, LC_50_, and LC_90_ values and the 95% confidence intervals of chlorfenapyr were calculated using the probit regression analysis program of SPSS version 25.0 (IBM company, Stanford, CA, USA).

We treated *P. marginatus* males in the subpupal and pupal stages as 3rd-instar nymphs to facilitate analysis of the life table parameters because females and males of *P. marginatus* have different instar durations. The raw data from the *P. marginatus* cohorts were analyzed using the TWOSEX-MSChart program (Hsin Chi, Taizhong, China) [28,29,30]. The variances and standard errors of developmental duration, longevity, fecundity, and population life table parameters were calculated using the bootstrap method, and significant differences were compared with the paired bootstrap test [31]. Population prediction was performed using the TIMING-MSChart program (Hsin Chi, Taizhong, China) [32]. Figures of the age-stage survival rate (*s_xj_*), age-specific survival rate (*l_x_*), age-specific fecundity (*m_x_*), age-specific maternity (*l_x_m_x_*), reproductive value (*v_xj_*), age-stage-specific life expectancy (*e_xj_*), and total population size were generated using Sigmaplot 12.2 software (Systat Software, Inc., San Jose, CA, USA).

The differences in the activities of the three antioxidative enzymes between the treatment and control groups were analyzed using *t*-test, and the graphs were plotted using GraphPad Prism 8.0.2 (GraphPad Software, Inc., San Diego, CA, USA).

## 3. Results

### 3.1. Toxicity of Chlorfenapyr on P. marginatus

The LC_30_, LC_50_, and LC_90_ of chlorfenapyr in 2nd-instar *P*. *marginatus* nymphs were 5.55 mg·L^−1^ (95% confidence limit, CL: 4.53–6.57 mg·L^−1^), 11.64 mg·L^−1^ (95% CL: 10.09–13.34 mg·L^−1^), and 71.17 mg·L^−1^ (95% CL: 56.69–94.56 mg·L^−1^), respectively.

### 3.2. Life History Traits

The sublethal concentration of chlorfenapyr had significant effects on the developmental time of the F_0_ generation of *P*. *marginatus* (Table 1). The duration of the 2nd-instar nymphs (*p* = 0.006) of the F_0_ individuals were significantly prolonged by chlorfenapyr treatment. Compared to the control group of mealybugs, the F_1_ individuals exposed to chlorfenapyr had a significant increase in the durations of the egg and 1st- and 2nd-instar nymphal stages (*p* *<* 0.001), while the duration of 3rd-instar nymphs and adult longevity of F_1_ individuals (*p* *<* 0.001) were significantly decreased. In the F_3_ generation of the treated group, the duration of the 1st-instar nymphs (*p* = 0.038) was significantly shortened, while the adult preoviposition period (APOP) (*p* < 0.001) was extended compared to the control group.

In F_3_ individuals from the chlorfenapyr treatment group, the durations of the egg and nymphal stages, and total preoviposition period (TPOP) were all significantly higher than they were in the F_0_ and F_1_ generations. The trend of F_3_ from the chlorfenapyr-treated group with higher duration than F_0_ and F_1_ was the same in the control group. These same parameters also differed significantly between the F_0_ and F_1_ generations (*p* < 0.001). However, the adult longevity and APOP in F_1_ individuals from the chlorfenapyr treatment group were significantly lower than they were in the F_0_ and F_3_ generations. The duration time of the egg, 1st- and 2nd-instars, and TPOP in the F_0_, F_1_, and F_3_ generations were all significantly different than equivalent values in the control group (*p* < 0.001), whereas no significant differences were observed for APOP in the different generations (Table 1). To sum up, the development durations of the preadult were prolonged.

### 3.3. Population Parameters

The transgenerational effects of chlorfenapyr (LC_30_) on the population parameters of the F_1_ and F_3_ generations were evaluated based on life table data (Table 2). Compared to the control group, the intrinsic rate of increase (*r*), finite rate of increase (*λ*), net reproductive rate (*R*_0_), and mean generation time (*T*) in the F_0_ generation of *P*. *marginatus* were not significantly affected by chlorfenapyr. Only the ratio of female adults to total individuals (*N_f/_N*) in the F_0_ generation was significantly decreased after the sublethal chlorfenapyr treatment (*p* = 0.026). However, other than the fecundity (*F*, *F_r_*), the values for the *r*, *λ*, *R*_0_, and ratio of reproductive females (*N_fr/_N_f_*) in the F_1_ were significantly decreased by chlorfenapyr. In contrast to the F_1_, in F_3_ individuals, only the *N_f/_N* was significantly increased in the chlorfenapyr treatment (*p* < 0.001) when compared with the control, with no significant differences found in the other F_3_ parameters.

The *r*, *λ, R*_0_, and *N_f/_N* values were significantly lower in the F_1_ than in the F_0_ and F_3_ generations, and the length of *T* in the F_1_ was significantly increased from the treated F_0_ to F_3_ generations (*p* < 0.05; Table 2). The *r*, *λ*, and *N_f/_N* values in the F_1_ were significantly lower than they were in the F_0_ and F_3_ generations, while the *T* and *F* values of the F_0_ were significantly lower than those of the control F_1_ to F_3_ generations. No significant difference was observed in the *N_fr/_N_f_* and *F* values among the F_0_, F_1_, and F_3_ generations of the chlorfenapyr treatment group.

### 3.4. Survival and Fecundity

**Figure 1:**
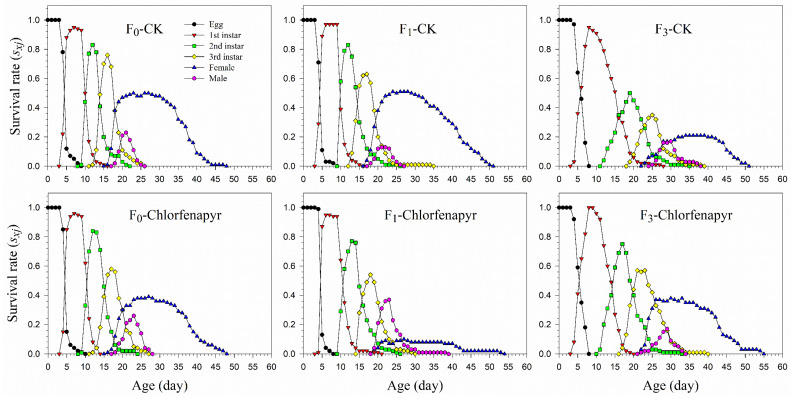
Age-stage-specific survival rate (*s_xj_*) of *P*. *marginatus* (F_0_, F_1_, and F_3_) exposed to LC_30_ of chlorfenapyr. CK represents the control.

The age-stage-specific survival rate (*s_xj_*) curves indicated the chance that a mealybug egg survives to age *x* and stage *j* and showed separately survival rate in different life stages of *P. marginatus* (Figure 1). Variations in peaks showed differences in the developmental stages. The probability of an egg surviving to the female adult stage in the F_1_ after being treated with chlorfenapyr was extremely low (0.14, 14 individuals) compared to eggs in the F_0_ (0.40, 40 individuals) and F_3_ (0.44, 44 individuals) generations.

**Figure 2:**
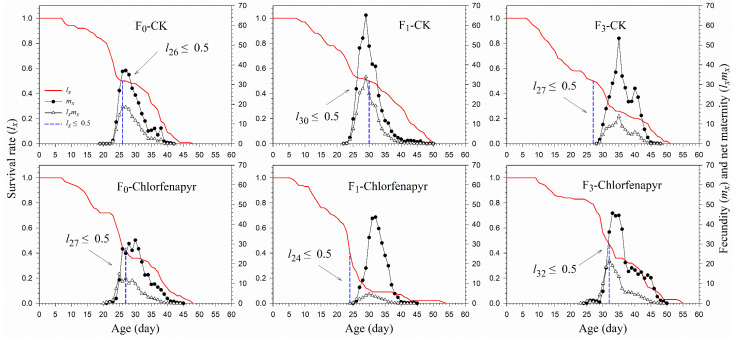
Survival rate (*l_x_*), fecundity (*m_x_*), and net maternity (*l_x_m_x_*) of *P*. *marginatus* (F_0_, F_1_, and F_3_) exposed to LC_30_ dose of chlorfenapyr. CK represents the control.

The age-specific survival rates (*l_x_*) of *P*. *marginatus* after different treatments are shown in Figure 2. The 50% survival rate of *P*. *marginatus* in the chlorfenapyr treatment groups occurred at ages 26, 24, and 32 d in the F_0_, F_1_, and F_3_ generations, respectively, and at ages 26, 30, and 27 d in F_0_, F_1_, and F_3_ of the control. Higher age-specific fecundity (*m_x_*) and net maternity (*l_x_m_x_*) curves were observed in the F_1_ of the control. Although the *m_x_* had a higher peak in the control (53.46 at 35 d) in the F_3_, the *l_x_* and *l_x_m_x_* values were lower than those in the chlorfenapyr treatment.

**Figure 3:**
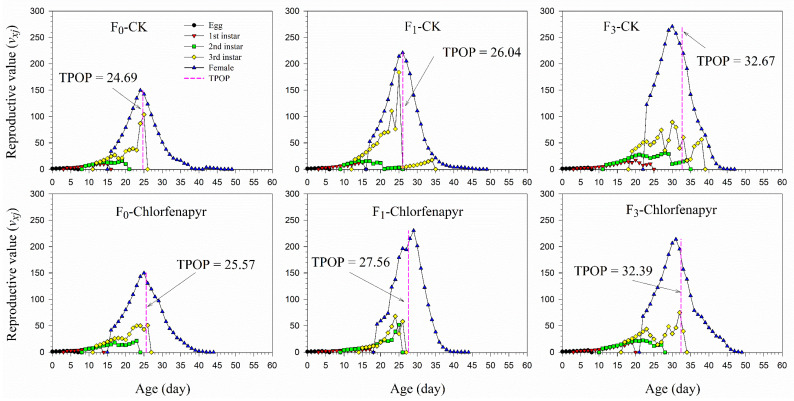
Age-stage-specific reproductive value (*v_xj_*) of *P*. *marginatus* (F_0_, F_1_, and F_3_) exposed to LC_30_ dose of chlorfenapyr. CK represents the control.

The age-stage-specific reproductive value (*v_xj_*) represents the contribution to future offspring of an individual from age *x* to stage *j* (Figure 3). The *v_xj_* value of the F_0_, F_1_, and F_3_ generations at age zero is a finite rate of increase (*λ*), i.e., 1.178, 1.114, and 1.157 in the chlorfenapyr treatment and 1.193, 1.202, and 1.140, respectively, in the control. The *v_xj_* value increased with age, with the *v_xj_* curve significantly increasing when the female adults emerged. In general, the maximum *v_xj_* values in different stages in the control were higher than those in the chlorfenapyr-treated group. The *v_xj_* peaks were close to the TPOP.

**Figure 4:**
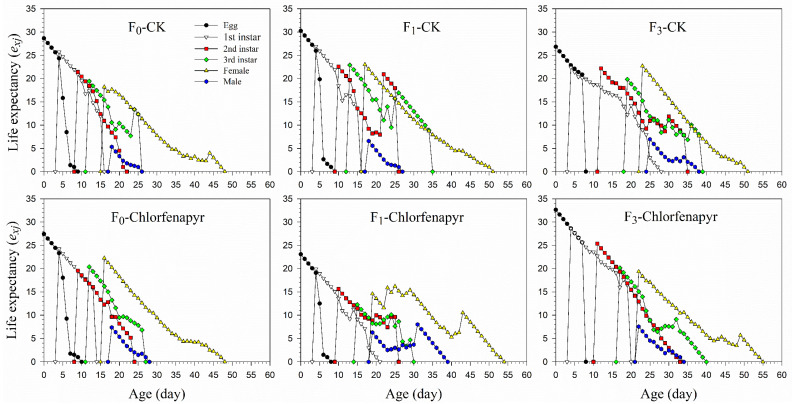
Age-stage-specific life expectancy (*e_xj_*) of *P*. *marginatus* (F_0_, F_1_, and F_3_) exposed to LC_30_ dose of chlorfenapyr. CK represents the control.

The age-stage-specific life expectancy (*e_xj_*) is the length of time that an individual of age *x* and stage *j* is expected to survive starting at age *x* (Figure 4). As expected, the highest *e_xj_* values for all tested generations occurred at the egg stage. The life expectancy of a newly laid egg of *P*. *marginatus* control females in generations F_0_, F_1_, and F_3_ was 28.65, 30.26, and 26.86 d, respectively, and 27.45, 23.08, and 32.62 d, respectively, in the chlorfenapyr treatment. Thus, the life expectancy was similar between the control and chlorfenapyr treatments in the F_0_ generation but significantly different in the F_1_ and F_3_ generations. The *exj* values were higher in the female than in the male adults in all of the generations.

### 3.5. Population Prediction

**Figure 5:**
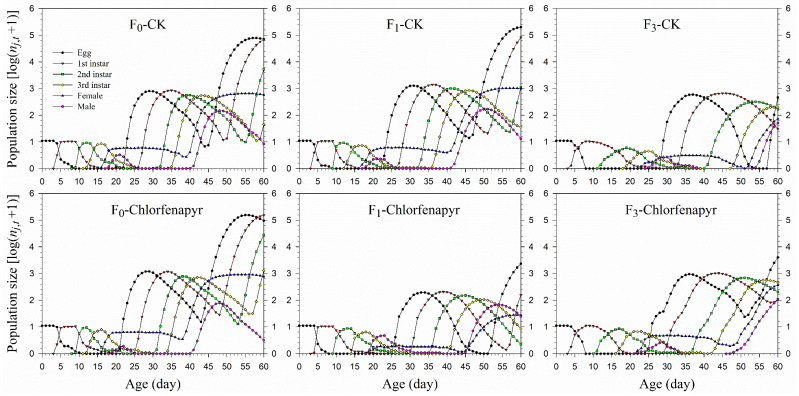
Population sizes of *P*. *marginatus* (F_0_, F_1_, and F_3_) exposed to LC_30_ dose of chlorfenapyr. CK represents the control.

**Figure 6:**
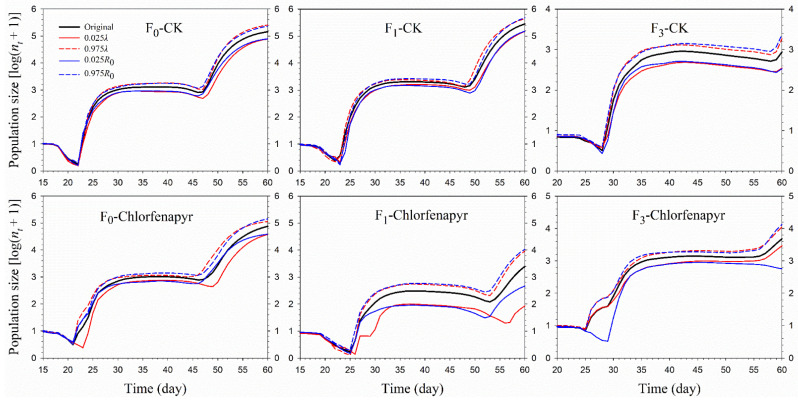
Population projection of *P. marginatus* (F_0_, F_1_, and F_3_) exposed to LC_30_ dose of chlorfenapyr by using the life tables based on the 2.5% and 97.5% percentiles of finite rate (*λ*) and net reproductive rate (*R*_0_). CK represents the control.

The population growth of *P. marginatus* treated with sublethal chlorfenapyr concentrations was simulated using the TIMING-MSChart program (Figure 5). Starting with an initial population of 10 newborn eggs, the F_0_, F_1_, and F_3_ populations of *P*. *marginatus* all developed to the fourth generation within 100 d in both the control and chlorfenapyr treatments. On the 100th d, the population with the largest number of individuals was the F_1_ of the control population (86,006,272), while the F_1_ in the chlorfenapyr treatment had the fewest number of individuals (262,695). Life tables from the 2.5th and 97.5th percentiles of *R*_0_ and *λ* can be projected to describe the variability of population growth. When the life tables of the 2.5th and 97.5th percentiles of *R*_0_ were used to predict the variability in population growth, the population size of *P*. *marginatus* in the F_1_ chlorfenapyr treatment was 7237 and 1,808,058, respectively. However, when the life tables of the 2.5th and 97.5th percentiles of *λ* were used, the population size of *P*. *marginatus* in the F_1_ chlorfenapyr treatment was 8659 and 1,633,191, respectively (Figure 6).

### 3.6. Detoxification Enzyme and Antioxidative Enzyme Activities

**Figure 7:**
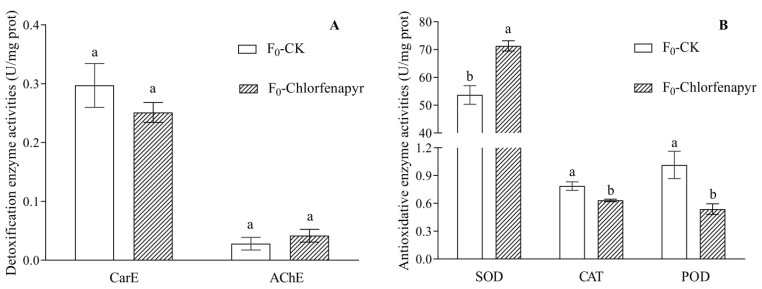
Effects of chlorfenapyr on detoxification enzyme (**A**) and antioxidative enzyme (**B**) activities of *P*. *marginatus* (F_0_-Chlorfenapyr) and F_0_-CK. Data are means ± SE of three biological replications; different letters above each bar indicate statistically significant difference by *t*-test (*p* < 0.05). CK represents the control, AchE is acetylcholinesterase, CarE is carboxylesterase, SOD is superoxide dismutase, CAT is catalase, and POD is peroxidase.

Compared with F_0_-CK, there were no significant differences of CarE and AchE activities in F_0_-Chlorfenapyr treated with LC_30_ (Figure 7A). The activities of three antioxidative enzymes, i.e., SOD, CAT, and POD, differed significantly between F_0_-Chlorfenapyr group and F_0_-CK group. The SOD activities in F_0_-Chlorfenapyr were significantly higher than that in F_0_-CK (*t* = 7.989, *df* = 4, *p* = 0.001), but the CAT and POD activities in F_0_-Chlorfenapyr were significantly lower than those in F_0_-CK (CAT: *t* = 5.642, *df* = 4, *p* = 0.005; POD: *t* = 5.220, *df* = 4, *p* = 0.006) (Figure 7B).

## 4. Discussion

At present, there is no effective pesticide for controlling *P*. *marginatus*. This study was conducted to provide needed information on chlorfenapyr use on *P*. *marginatus*. No phytotoxicity has been observed when chlorfenapyr was used at recommended doses in the field (British Crop Production Council Pesticide Manual). In this study, the LC_90_ of chlorfenapyr on 2nd-instar nymphs was 71.17 mg·L^−1^ after 48 h of exposure. Approximately 60% of the field-recommended minimum dose (90 g a.i.·ha^−1^) can achieve 90% control of *P. marginatus*, requiring a much lower dose than the commonly used field-recommended dose. The findings presented here suggest that chlorfenapyr can be an effective pesticide for control of *P. marginatus*.

In addition, insect populations are frequently exposed to low and sublethal concentrations of insecticides in the field because of heterogeneous spatial coverage on plants or environmental degradation of pesticide [33]. Sublethal effects, such as prolonged development and reduced longevity and fecundity, are usually observed in many pests after exposure to sublethal concentrations of insecticides [19,20]. In our study, chlorfenapyr treatments prolonged the durations of the nymphal instars and adult longevity in the F_0_ generation. The insecticide-induced effects can be transgenerationally inherited [34]. In our study, the durations of the egg and young nymphal stages were increased in the F_1_ generation, but the duration of older nymph stages was reduced. In the subsequent F_3_ generation, after chlorfenapyr treatment, the duration of the 1st instar was shortened, and APOP was lengthened. Sublethal and transgenerational effects of several insecticides have been reported in other insects. For example, flupyradifurone affected the duration and survival rate of the F_1_ and F_2_ generations of *Myzus* *persicae* (Sulzer) (Hemiptera: Aphididae) [35], while the LC_30_ of six different insecticides had significant negative impacts on the life-history parameters of *Bactrocera* *dorsalis* (Hendel) (Diptera: Tephritidae) that led to reduced adult longevity and fecundity in the F_0_ generation and reduced fertility and survival in the F_1_ [36]. Our results provide evidence that the sublethal concentrations of chlorfenapyr do have significant sublethal and transgenerational effects on *P*. *marginatus*, and the negative sublethal effects may increase the biological fitness cost.

The indicators *r*, *R*_0_, and *λ* are important for evaluating insect populations [18,37]. The results in this study showed that treatments with sublethal doses of chlorfenapyr resulted in significantly decreased *r*, *R*_0_, and *λ* values in the F_1_ although no differences were observed in these parameters in the F_0_. Similarly, these demographic parameters were also significantly decreased following treatments with an LC_50_ dosage of triflumezopyrim in the F_5_ generation of *Laodelphax striatellus* Fallén (Hemiptera: Delphacidae) [38] and treatment with LC_5_ and LC_10_ dosages of chlorfenapyr in *Bradysia odoriphaga* Yang and Zhang (Diptera: Sciaridae) [39].

Insecticide-induced hormesis that inhibits or increases the fecundity of insects has been reported in many studies [40,41]. In this study, we found that the LC_30_ concentration of chlorfenapyr effectively increased fecundity in the F_0_ but inhibited the population recovery of *P*. *marginatus* in the subsequent F_1_ and F_3_ generations. This effect was likely related to the ratio of female adults to total individuals (*N_f_*/*N*) and the ratio of reproductive females (*N_fr_/N_f_*) in the treatment group being significantly higher than that in the control in the F_1_ and F_3_ generations (Table 1). Females play a decisive role in the growth of insect populations; therefore, a significant decrease in the proportion of females will result in a significant decrease in the reproductive rate of *P*. *marginatus*.

Sublethal doses not only affect the growth, development, and reproduction of pests but also induce the changes of enzyme activity in the insect body, which is conducive to the accumulation and development of resistance [42]. The results of the present study showed that the activities of AChE and CarE were not affected by the LC_30_ of chlorfenapyr, but the activities of SOD were significantly activated, while CAT and POD were significantly decreased. The antioxidative enzymes SOD, CAT, and POD protect the cells from injury in organisms [20]. SOD can convert free superoxide anion radicals into hydrogen peroxide, and CAT and POD break the hydrogen peroxide into water and oxygen [43]. This finding suggested that SOD, CAT, and POD may play an important role in the self-protection of *P*. *marginatus* to protect against chlorfenapyr.

In summary, the sublethal exposure to parental papaya mealybugs led to a significant increase in the durations of the egg and nymph stages and reduced the longevity, APOP, and population parameters *r*, *R*_0_, and *λ* in the F_1_ generation, but recovery to the control level occurred in the F_3_ generation. That is, lower concentrations of chlorfenapyr can potentially be used to control papaya mealybugs in the field at less than the recommended field dose. While our study may indicate that lower concentrations are possible, other aspects, such as the efficacy of the application, location of the pest on the plant, residue, etc., should be considered and evaluated when determining the optimal dosage of pesticide. Our study demonstrated that chlorfenapyr-mediated sublethal effects occur in at least two successive generations of *P*. *marginatus*. Therefore, when reapplying chlorfenapyr at an appropriate time, it is necessary to comprehensively consider this result, pest damage, natural enemies, and economic threshold, etc., in order to suppress *P*. *marginatus* population and prevent its outbreak. Low doses and prolonged application intervals will help to delay the development of insecticide resistance and reduce the adverse effects of pesticides on the environment and the human population.

## Figures and Tables

**Table 1:** Duration of each development stage, longevity, APOP, TPOP and oviposition days of *P*. *marginatus* in different generation exposed to LC_30_ of chlorfenapyr.

Stage	Generation	Control		LC_30_	
		*n*	Mean ± SE	*n*	Mean ± SE
Egg (d)	F_0_	-	-	-	-
	F_1_	97	4.78 ± 0.06 bB	95	5.07 ± 0.03 aB
	F_3_	100	6.23 ± 0.12 aA	100	6.04 ± 0.12 aA
1st instar (d)	F_0_	-	-	-	-
	F_1_	90	5.88 ± 0.12 bB	82	6.11 ± 0.15 aB
	F_3_	62	10.08 ± 0.31 aA	84	8.40 ± 0.16 bA
2nd instar (d)	F_0_	85	3.93 ± 0.12 bC	74	4.74 ± 0.17 aC
	F_1_	73	4.45 ± 0.14 bB	67	5.60 ± 0.25 aB
	F_3_	49	6.57 ± 0.37 aA	82	6.35 ± 0.27 aA
3rd instar (d)	F_0_	81	4.28 ± 0.08 aB	68	4.41 ± 0.09 aC
	F_1_	68	4.97 ± 0.16 aA	61	4.79 ± 0.10 bB
	F_3_	45	5.04 ± 0.28 aA	68	5.21 ± 0.17 aA
Adult longevity (d)	F_0_	81	12.89 ± 0.93 aB	68	12.78 ± 0.99 aA
	F_1_	68	16.87 ± 1.09 aA	61	6.62 ± 0.84 bB
	F_3_	45	10.96 ± 1.11 aB	68	11.94 ± 1.04 aA
APOP (d)	F_0_	49	6.14 ± 0.36 aA	35	6.11 ± 0.25 aA
	F_1_	51	5.96 ± 0.25 aA	9	5.11 ± 0.42 aB
	F_3_	21	5.48 ± 0.48 bA	36	7.11 ± 0.62 aA
TPOP (d)	F_0_	49	24.69 ± 0.38 aC	35	25.57 ± 0.40 aC
	F_1_	51	26.04 ± 0.41 aB	9	27.56 ± 0.84 aB
	F_3_	21	32.67 ± 0.78 aA	36	32.39 ± 0.72 aA
Oviposition days (*O_d_*) (d)	F_0_	49	7.37 ± 0.41 aB	35	8.37 ± 0.59 aA
	F_1_	51	9.31 ± 0.61 aA	9	9.11 ± 1.54 aA
	F_3_	21	8.43 ± 0.61 aA	36	8.19 ± 0.69 aA

Standard errors (SE) were estimated by using the bootstrap technique with 100,000 re-samplings. Significant differences at *p* < 0.05 between two different treatments and generations were compared with the paired bootstrap test implemented in the TWOSEX-MSChart program. The lower-case letters show significant differences between control and chlorfenapyr treatments in the same generation, while the capital letters indicate significant differences among the F_0_, F_1_, and F_3_ generations within the same treatment (*p* < 0.05).

**Table 2:** Sublethal effects of chlorfenapyr on the population parameters of *P*. *marginatus* in different generations.

Population Parameter	Generation	Control	LC_30_
Intrinsic rate of increase, *r* (day^−1^)	F_0_	0.18 ± 0.01 aA	0.16 ± 0.01 aA
	F_1_	0.18 ± 0.01 aA	0.11 ± 0.02 bC
	F_3_	0.13 ± 0.01 aB	0.15 ± 0.01 aB
Finite rate of increase, *λ* (day^−1^)	F_0_	1.19 ± 0.01 aA	1.18 ± 0.01 aA
	F_1_	1.20 ± 0.01 aA	1.11 ± 0.02 bC
	F_3_	1.14 ± 0.01 aB	1.16 ± 0.01 aB
Net reproductive rate, *R*_0_ (offspring)	F_0_	137.10 ± 22.22 aB	109.48 ± 18.34 aA
	F_1_	212.85 ± 29.45 aA	31.62 ± 13.05 bB
	F_3_	96.91 ± 23.10 aB	148.14 ± 27.12 aA
Mean generation time, *T* (day)	F_0_	27.77 ± 0.40 aC	28.65 ± 0.48 aC
	F_1_	29.04 ± 0.25 bB	31.25 ± 0.52 aB
	F_3_	34.67 ± 0.65 aA	34.22 ± 0.48 aA
Ratio of female adults in total individuals (*N_f_*/*N*)	F_0_	0.56 ± 0.05 aA	0.40 ± 0.05 bA
	F_1_	0.54 ± 0.05 aA	0.14 ± 0.03 bB
	F_3_	0.22 ± 0.04 bB	0.44 ± 0.05 aA
Ratio of reproductive females (*N_fr_/N_f_*)	F_0_	0.88 ± 0.04 aA	0.88 ± 0.05 aA
	F_1_	0.94 ± 0.03 aA	0.64 ± 0.13 bA
	F_3_	0.95 ± 0.05 aA	0.82 ± 0.06 aA
Fecundity (*F*) (eggs/female)	F_0_	244.82 ± 33.28 aB	273.70 ± 31.78 aA
	F_1_	394.17 ± 41.19 aA	225.86 ± 77.39 aA
	F_3_	440.50 ± 66.18 aA	336.68 ± 49.23 aA
Fecundity (*F_r_*) (eggs/reproductive female)	F_0_	279.80 ± 35.31 aB	312.80 ± 31.05 aA
	F_1_	417.35 ± 41.36 aA	351.33 ± 98.12 aA
	F_3_	461.48 ± 65.83 aA	411.50 ± 52.53 aA

Standard errors (SE) were estimated by using the bootstrap technique with 100,000 re-samplings. Significant differences at *p* < 0.05 between two different treatments and generations were compared with the paired bootstrap test implemented in the TWOSEX-MSChart program. The lower-case letters show significant differences between control and chlorfenapyr treatments in the same generation, while the capital letters indicate the significant differences among the F_0_, F_1_, and F_3_ generations within the same treatment (*p* < 0.05).

## Data Availability

Data are contained within the article.
